# Total retroperitoneal lipectomy improves prognosis in patients with primary retroperitoneal liposarcoma: a comparative study

**DOI:** 10.3389/fonc.2024.1488143

**Published:** 2024-12-04

**Authors:** Haicheng Gao, Shibo Liu, Wenjie Li, Boyuan Zou, Chengli Miao

**Affiliations:** Department of Retroperitoneal Tumor Surgery, Peking University International Hospital, Beijing, China

**Keywords:** retroperitoneal liposarcoma, total retroperitoneal lipectomy, complete resection, prognosis, complication

## Abstract

**Background:**

Retroperitoneal liposarcoma (RPLS) is the most common soft tissue sarcoma originating in the retroperitoneal space. Although surgery is the standard treatment, recurrence remains frequent. In this study, we aimed to explore the safety and efficacy of total (ipsilateral) retroperitoneal lipectomy (TRL) compared to traditional complete resection (CR) for primary RPLS.

**Methods:**

We retrospectively analyzed patients with primary RPLS treated at our center between January 2014 and December 2020. Univariate and multivariable Cox regression analyses assessed the impact of demographic, operative, and clinicopathological variables on recurrence-free survival (RFS) and overall survival (OS). Kaplan-Meier plots illustrated RFS and OS, and the log-rank test compared time-to-event distributions.

**Results:**

A total of 81 patients were included in the final analysis: 37 in the CR group and 44 in the TRL group. Demographic and clinicopathologic parameters were comparable between the two groups. Post-operative morbidity occurred in 30.9% of cases, with 15 (40.5%) in the CR group and 10 (22.7%) in the TRL group (P=0.086). There were 9 cases of severe complications at grade 3 or higher, with 5 cases in the CR group and 4 cases in the TRL group. There was no significant difference between the two groups (P=0.314). The TRL group demonstrated improved RFS and OS, particularly among dedifferentiated liposarcoma (DDLS) patients.

**Conclusions:**

Total retroperitoneal lipectomy (TRL) appears to be a safe procedure that enhances survival outcomes in patients with primary RPLS. Further studies are needed to validate these findings.

## 1 Introduction

Retroperitoneal liposarcoma (RPLS) is the most prevalent malignancy among retroperitoneal sarcomas (RPS), which accounts for approximately 0.15% of all adult cancers and has an incidence of 0.5–1 case per 100,000 (1, 2). RPLS poses significant challenges for treatment due to its potential of adjacent organ involvement and frequent recurrence. The role of radiation and systemic therapy in RPLS is not well defined, and surgery is currently the only potentially curative treatment choice (3, 4). Macroscopic complete resection (CR) combined with the resection of involved adjacent organs has been recommended for the treatment of RPLS. However, local recurrence remains common (40–85%) (4).

The inability to achieve a true R0 resection with the susceptive microscopic involvement of adjacent organs, structures, and surfaces might contribute to the high rate of postoperative recurrence in RPLS (5). Multiple satellite tumor foci may exist in the perceived normal adipose tissue that can be separated from the visible tumor (5, 6). Complications arising from recurrence, such as ileus, cachexia, and multiple organ dysfunction are the main cause of tumor related death. Many surgical oncologists recommend extended resection for RPLS to improve prognosis based on experience or clinical research (7, 8). However, Controversy exists over whether normal adipose tissue adjacent to the tumor should be removed in addition to combined resection of organs invaded by the tumor and abnormal adipose tissue.

In this regard, we reviewed primary RPLS patients treated with CR or TRL in our department, a center focused on the treatment of retroperitoneal tumors, to further clarify the effect of TRL in treating primary RPLS compared with traditional CR.

## 2 Materials and methods

### 2.1 Patient selection

Patients with unilateral primary RPLS who underwent resection with curative intent between January 2014 and December 2020 were identified from prospectively maintained sarcoma databases at our hospital. Only patients with well-differentiated liposarcoma (WDLPS) or dedifferentiated liposarcoma (DDLPS) who were treated with R0/R1 resection were included in this study. Patients with central (mesenteric) or primarily pelvic tumors, grossly incomplete (R2) resection, missing clinical information or history of other malignancies were excluded from this study.

Electronic medical records were retrieved to extract data on the following variables: (I) preoperative variables [i.e., age, gender), preoperative hemoglobin (Hb), albumin (ALB), receipt of neoadjuvant or adjuvant therapy, tumor size (maximum diameter), tumor site, and number of tumors (unifocal vs. multifocal); (II) intraoperative variables [i.e., type of surgery (TRL vs. CR), organs resected, operation duration, and estimated blood loss]; and (III) postoperative variables [i.e., histologic subtype, length of hospital stay, complications according to Clavien-Dindo classification, dates of recurrence, and death]. To assess these variables, patients’ medical history, radiologic imaging, operative notes, and pathological reports were reviewed and integrated by experienced multidisciplinary sarcoma specialists. A unifocal tumor was defined as 1 solitary tumor in the retroperitoneum, while multifocal tumors were defined as the presence of 2 or more non-contiguous tumors in the retroperitoneum, as determined by preoperative computed tomography (CT) scans and confirmed by intraoperative findings. Patients who had both WDLPS and DDLPS components in their tumors were classified as DDLPS.

### 2.2 Standard of CR and TRL

**Figure 1:**
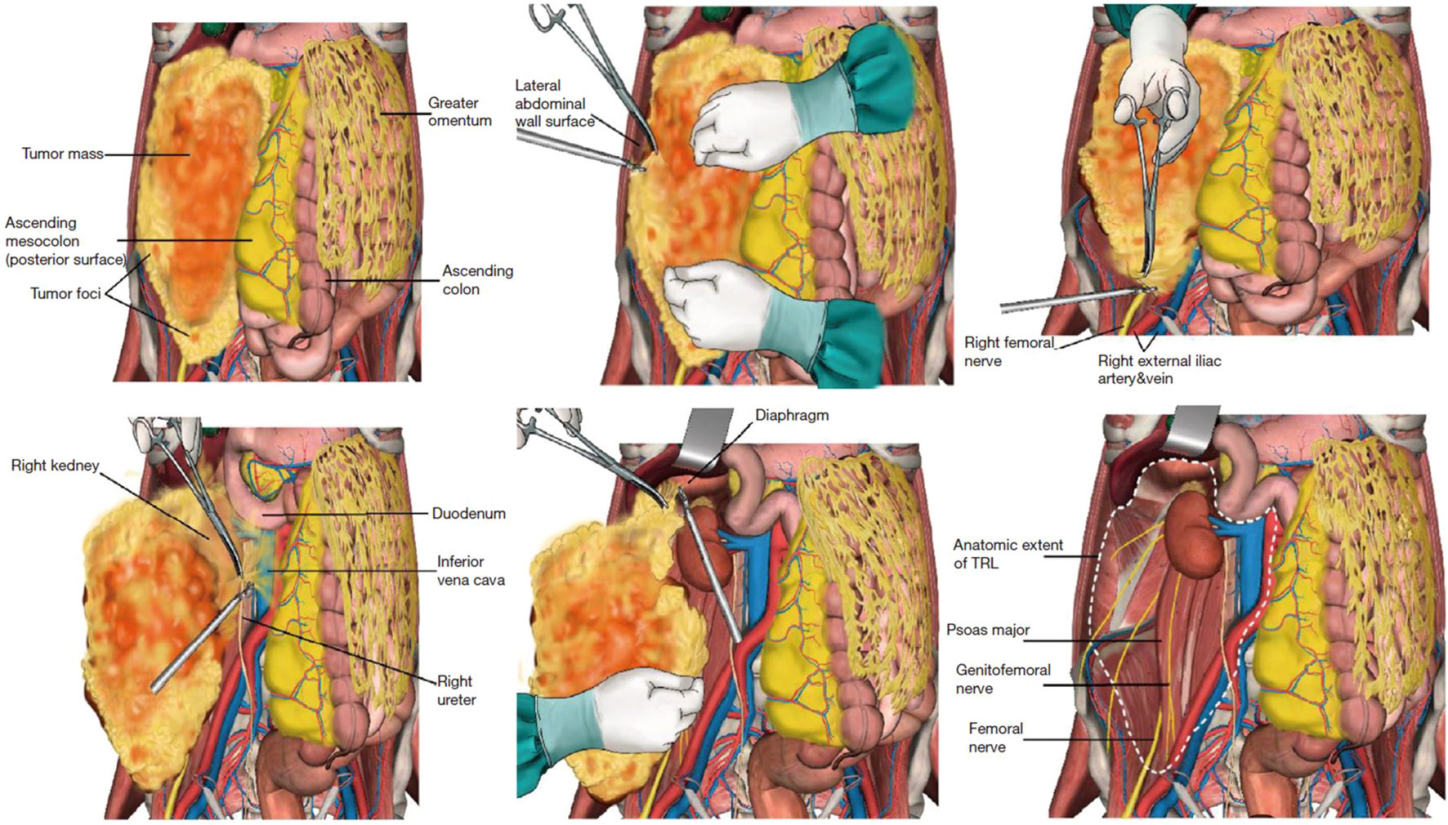
Schematic diagram of the six borders in the TRL procedure. Reproduced with permission from Miao et al. (8), licensed under CC BY-NC-ND 4.0.

CR was defined as the surgical resection of the total tumor mass with grossly negative margins (R0/R1). To achieve this goal, *en-bloc* resection of the tumor with grossly involved adjacent organs and/or major vessels was carried out. In TRL, in addition to CR, all the ipsilateral retroperitoneal adipose tissue was removed, regardless of normal or abnormal fat. The anatomic extent of lipectomy in TRL was demarcated by the following 6 borders: anterior (the posterior surface of abdominal viscera); posterior (the psoas, iliopsoas, and other muscle surfaces); superior (the diaphragm surface); inferior (the iliac vascular surface); medial [the inferior vena cava surface (to the right) or abdominal aorta surface (to the left)]; and lateral (the lateral abdominal wall surface at mid-auxiliary line level). The aforementioned borders are shown in **Figure 1**.

### 2.3 Follow-up

Postoperative baseline CT scans were performed to ensure the complete removal of gross visible adipose tissue in all RPLS patients. Patients continued to receive CT scans of the abdomen/pelvis every 3 months for 2 years, and then every 6 months for 5 years as recommended by the National Comprehensive Cancer Network (NCCN, United States of America) and The Trans-Atlantic Retroperitoneal Sarcoma Working Group (TARPSWG). For patients with high-grade DDLPS tumors, contrast-enhanced CT of the chest was added as a form of surveillance imaging.

All patients were followed up by outpatient records or telephone conversations.

### 2.4 Statistical analysis

The TRL- and CR-related parameters were compared by independent sample t-tests for the numerical variables and Wilcoxon rank-sum test for the categorical variables. Recurrence-free survival (RFS) and overall survival (OS) were defined as the time from the date of surgery to the date of recurrence, or to death/last at follow-up, respectively. Survival curves were obtained by means of Kaplan-Meier plots to estimate the RFS and OS. The log-rank test was used to compare the survival outcomes. To identify the patient population that would benefit the most from TRL, univariable and multivariable Cox proportional-hazards regression models were used. Variables with P-values less than 0.1 in univariate Cox regression analysis are included in multivariate Cox regression analysis. All the statistical analyses were carried out using SPSS software (version 22.0), and a P value <0.05 was considered statistically significant.

## 3 Results

### 3.1 Baseline characteristics and surgery details

In total, 81 patients met the inclusion criteria for this study and were included in the final study, with 37 patients in the CR group and 44 patients in the TRL group. Clinicopathologic features of patients were listed in **Table 1**. As shown, there was no significant difference in gender, age, tumor size, tumor location, number of tumors, preoperative hemoglobin and albumin, adjuvant therapy or surgical details.

**Table 1:** Baseline characteristics of the 81 patients with primary retroperitoneal liposarcoma.

Variable		ALL	CR	TRL	P value
Gender					0.111
	Male	47	25	22	
	Female	34	12	22	
Age, years			54.7 ± 12.1	52.4 ± 12.7	0.409
Tumor size, cm					0.498
	≤10cm		6	4	
	10~20cm		10	10	
	≥20cm		21	30	
Location					0.934
	center		18	21	
	Right		19	23	
Number of tumors					0.570
	Unifocal		20	21	
	Multifocal		17	23	
Hemoglobin, g/L			118.7 ± 20.5	120.7 ± 22.2	0.681
Albumin, g/L			34.3 ± 5.6	36.5 ± 6.0	0.088
Adjuvant therapy					0.700
	Yes		4	6	
	No		33	38	
Combined evisceration					0.089
	Yes		23	19	
	No		14	25	
Operation duration, minutes			309 ± 129	315 ± 89	0.813
Estimated blood loss, ml, IQR		800 (400, 1625)	1000 (450, 1900)	650 (400, 1500)	0.152
Histology					0.371
	WDLS		14	21	
	DDLs		23	23	
Post-operative complications					0.084
	Yes		15	10	
	No		22	34	
Severe complications					0.314
	Yes		5	4	
	No		32	41	
Length of hospital stay, day			26.0 ± 14.5	22.0 ± 8.8	0.129

### 3.2 Post-operative morbidity

A total of 25 patients (30.9%) had postoperative complications. Among them, there were 15 cases (40.5%) in the CR group and 10 cases (22.7%) in the TRL group, with no significant difference between the two groups (P=0.086). Severe complications of grades 3 and 4 occurred in a total of 8 cases, with a rate of 9.9%. Among these, 5 cases (13.5%) were in the CR group and 3 cases (6.8%) were in the TRL group, with no significant difference between the groups (P=0.317). There were no perioperative deaths, and no readmissions within 30 days after discharge.

### 3.3 Follow-up results

Patients were followed up by telephone or outpatient visits. The average follow-up duration was 61.5 months (range: 11-107). No patients were lost to follow-up, and all patients were included in the final survival analysis.

#### 3.3.1 RFS

**Figure 2:**
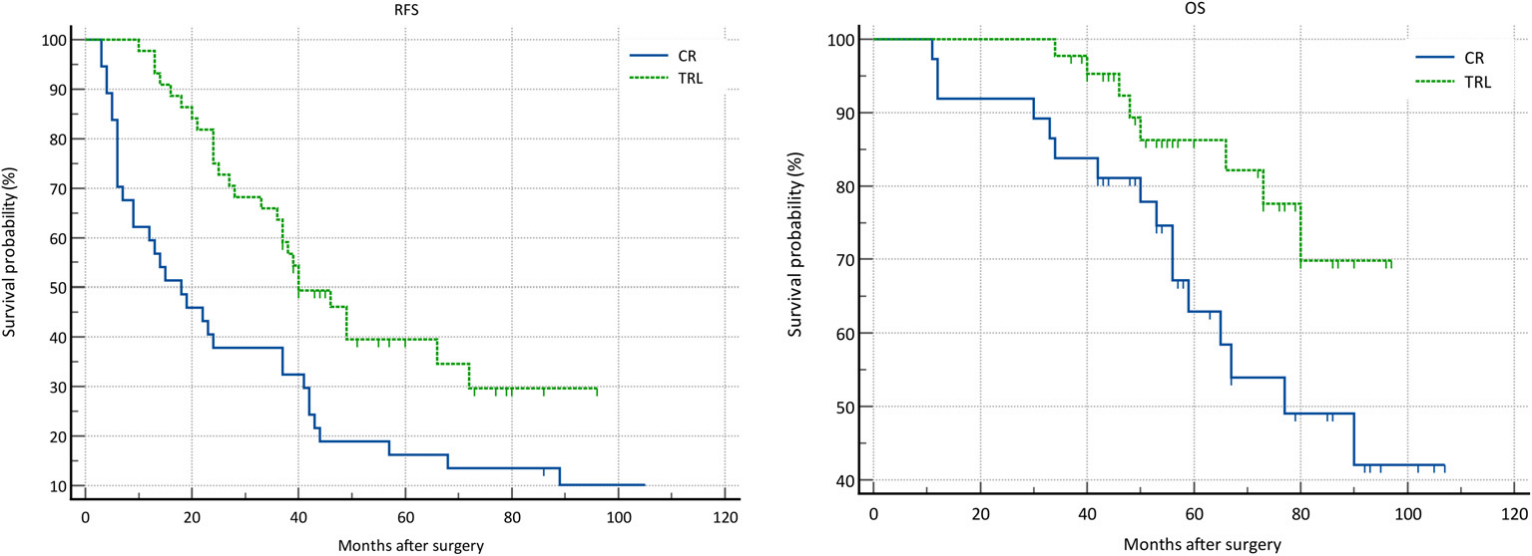
Comparison of RFS (P=0.002) and OS (P=0.030) Between CR and TRL group in all patients.

**Figure 3:**
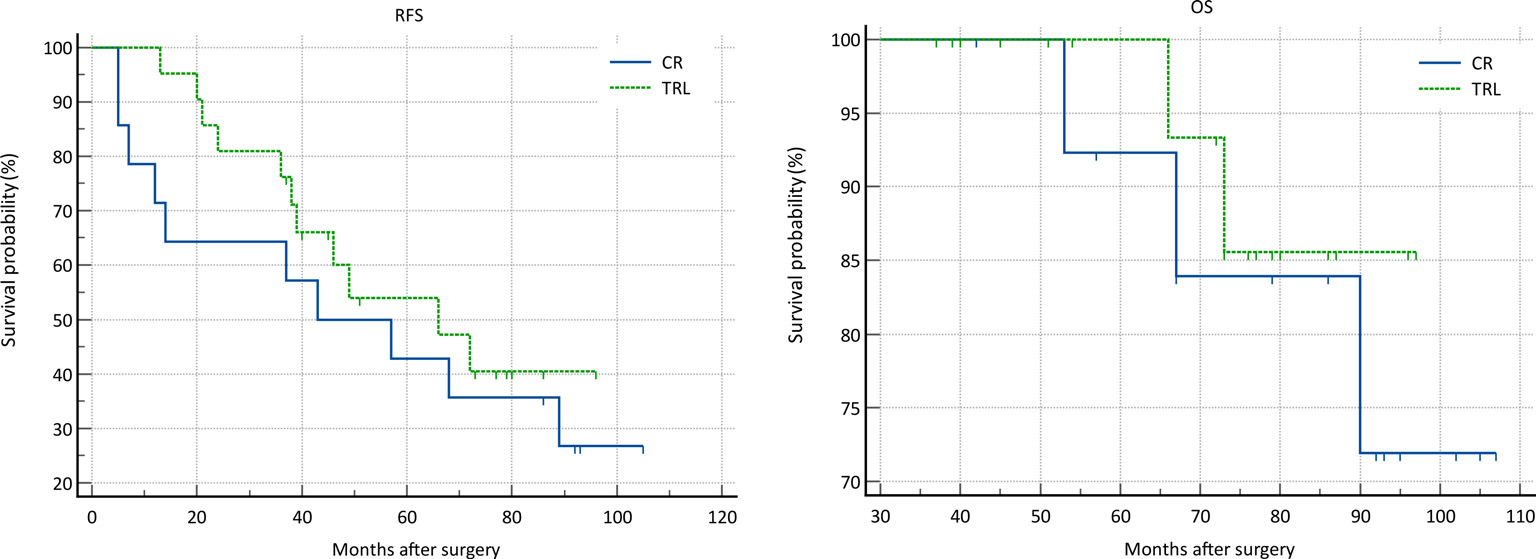
Comparison of RFS (P=0.443) and OS (P=0.654) between CR and TRL group in WDLS patients.

**Figure 4:**
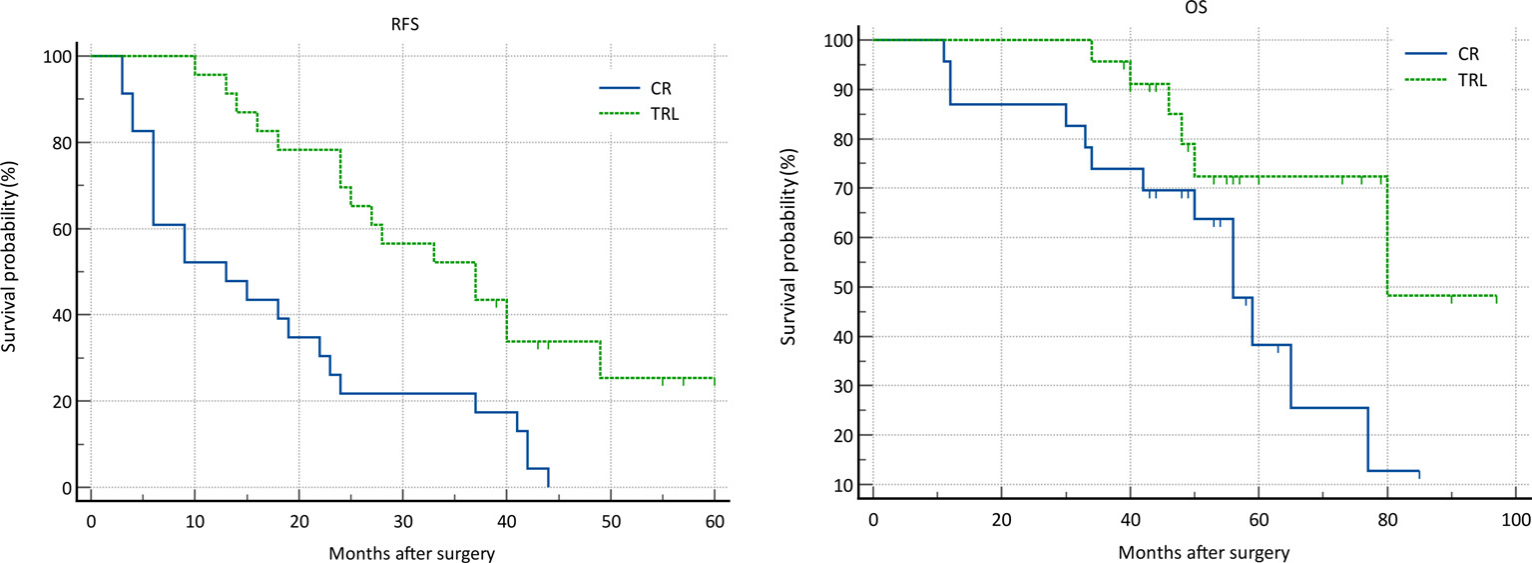
Comparison of RFS (P<0.001) and OS (P=0.033) between CR and TRL group in DDLS patients.

**Figure 5:**
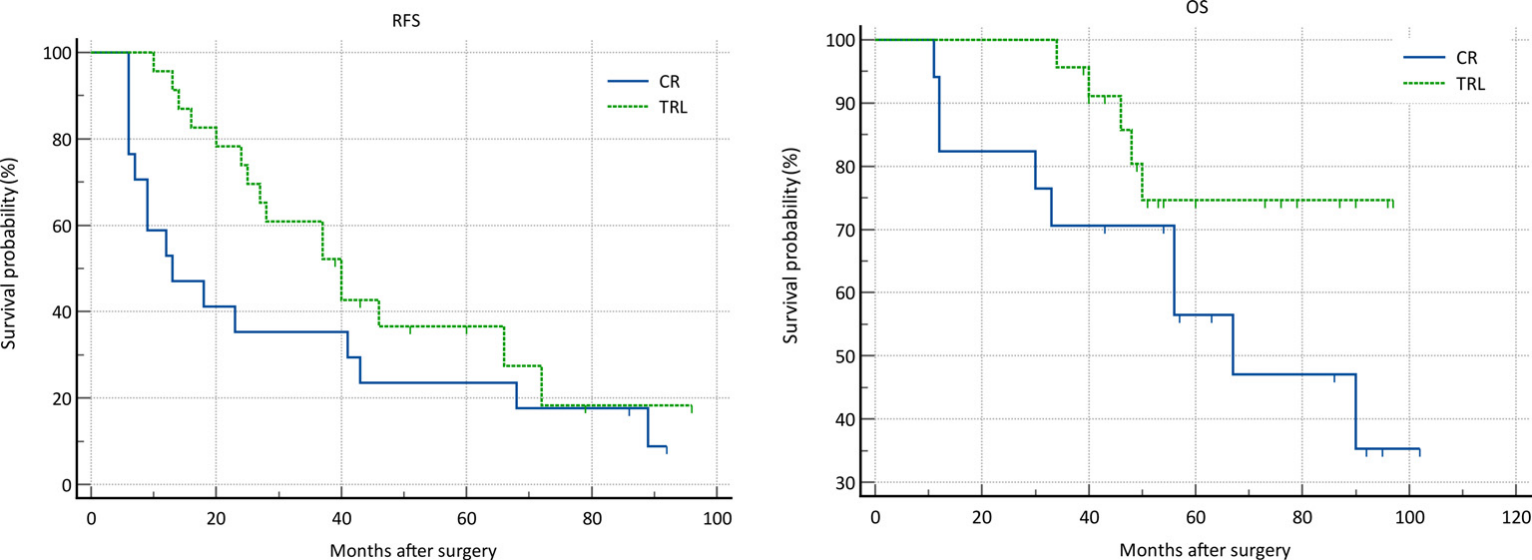
Comparison of RFS (P=0.123) and OS (P=0.082) between CR and TRL group in multifocal tumor patients.

**Figure 6:**
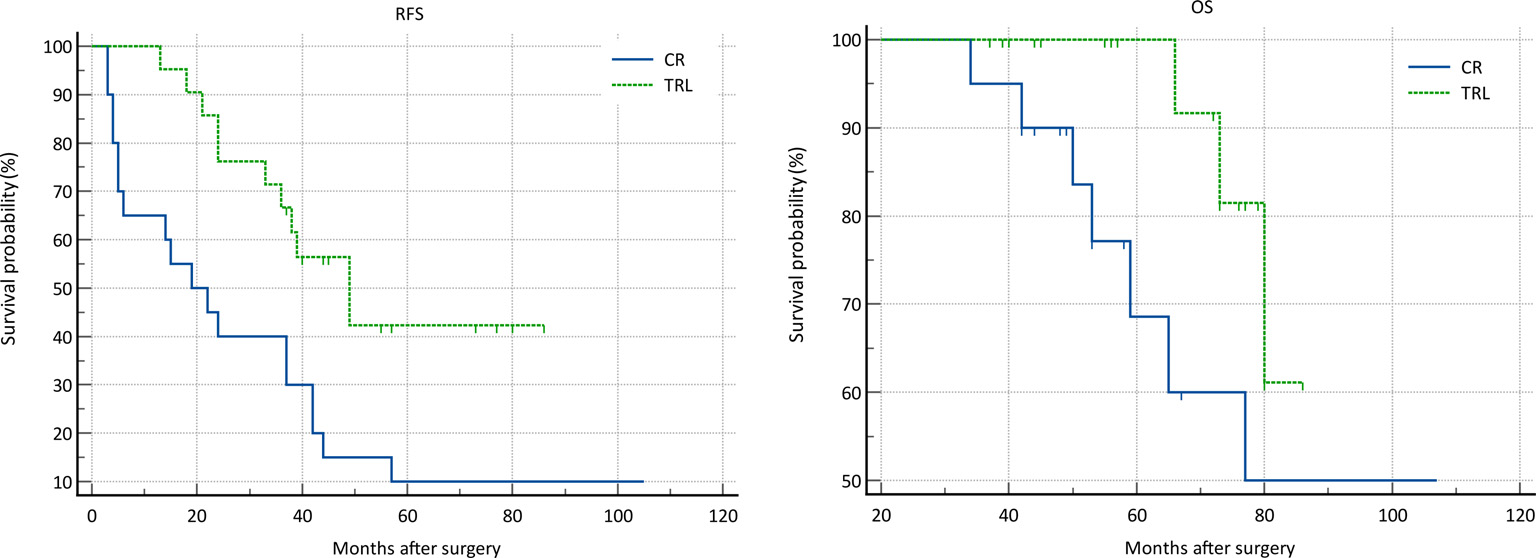
Comparison of RFS (P=0.004) and OS (P=0.119) between CR and TRL group in unifocal tumor patients.

In the entire patient cohort, TRL group patients had significantly better recurrence-free survival (RFS) compared to CR group patients (P=0.002). The 1-year RFS rates were 80.2% and 59.5%, respectively (P<0.001), while the 3-year RFS rates were 46.9% and 32.4% (P<0.001). Subgroup analysis revealed that TRL improved RFS in DDLS patients (P<0.001), whereas in WDLS patients, there was no significant difference in RFS between TRL and CR groups (P=0.443). Additionally, TRL improved RFS in unifocal patients (P=0.004), while for multifocal patients, there was no significant difference in RFS between the two surgical approaches (P=0.123). (see **Figures 2**–**6**) In multivariate analysis, histology and post-operative complications were confirmed as independent factors correlated with tumor recurrence (**Table 2**).

**Table 2:** Univariable and multivariable analysis of associations between clinicopathological factors and RFS.

Variable	Univariate analysis			Multivariate analysis		
	HR	95%CI	P value	HR	95%CI	P value
Gender (female vs. male)	0.7018	0.4179-1.1785	0.177	–	–	–
Age, year	1.0025	0.9823-1.0230	0.811	–	–	–
Tumor size, cm						
10~20 vs. <10	1.1065	0.4668-2.6224	0.818	–	–	–
>20 vs. <10	1.0349	0.4785-2.2385	0.931	–	–	–
Location (left vs. right)	0.5610	0.3333-0.9441	0.028	0.5873	0.3396-1.0033	0.051
Number of tumors (multifocal vs. unifocal)	1.1070	0.6666-1.8384	0.694	–	–	–
Hemoglobin, g/L	0.9951	0.9832-1.0073	0.434	–	–	–
Albumin, g/L	0.9621	0.9206-1.0055	0.091	1.0001	0.9518-1.0509	
Adjuvant therapy (yes vs. no)	1.7815	0.8639-3.6734	0.142	–	–	–
Combined evisceration (yes vs. no)	1.8467	1.0320-3.0931	0.019	1.2497	0.6694-2.3331	0.484
Operation duration, min	1.0023	0.9997-1.0050	0.086	1.0000	0.9969-1.0031	0.991
Estimated blood loss, ml	1.0002	1.0000-1.0003	0.138	–	–	–
Histology						
DDLS vs. WDLS	2.9811	1.6572-5.3627	<0.001	2.9121	1.5603-5.4351	0.001
Post-operative complications (yes vs. no)	2.5136	1.4687-4.3021	0.001	2.4418	1.0992-5.4239	0.028
Severe complications (yes vs. no)	1.4190	0.6067-3.3188	0.440	–	–	–
Length of hospital stay, day	1.0193	0.9978-1.0412	0.094	0.9870	0.9561-1.0189	0.419
TRL vs. CR	0.4588	0.2746-0.7665	0.003	0.4631	0.27310.7850	0.004

#### 3.3.2 OS

In the entire patient cohort, TRL group patients had significantly better overall survival (OS) compared to CR group patients (P=0.030). The 1-year OS rates were 96.3% and 91.9%, respectively (P=0.015), while the 3-year OS rates were 91.4% and 83.8% (P=0.026). Subgroup analysis revealed that TRL improved OS in DDLS patients (P=0.033), while in WDLS patients, there was no significant difference in OS between the two surgical approaches (p=0.654). For both multifocal and unifocal patients, there was no significant difference in OS, with P-values of 0.082 and 0.119, respectively. (see **Figures 2**–**6**) In multivariate analysis, only histology was confirmed as independent factors correlated with tumor related death (**Table 3**).

**Table 3:** Univariate and multivariate analysis of associations between clinicopathological factors and OS.

Variable	Univariate analysis			Multivariate analysis		
	HR	95%CI	P value	HR	95%CI	P value
Gender (female vs. male)	0.2949	0.1099-0.7911	0.015	0.4936	0.1631-1.4942	0.212
Age, year	1.0260	0.9914-1.0618	0.142	–	–	–
Tumor size, cm				–	–	–
10~20 vs. <10	0.5714	0.1573-2.0755	0.395	–	–	–
>20 vs. <10	0.5867	0.1898-1.8139	0.355	–	–	–
Location (left vs. right)	0.6901	0.3014-1.5800	0.374	–	–	–
Number of tumors (multifocal vs. unifocal)	1.5181	0.6712-3.4337	0.313	–	–	–
Hemoglobin, g/L	0.9719	0.9536-0.9906	0.003	0.9754	0.9467-1.0051	0.103
Albumin, g/L	0.8966	0.8381-0.9592	0.002	1.0171	0.9066-1.1410	0.773
Adjuvant therapy (yes vs. no)	1.8869	0.6398-5.5652	0.250	–	–	–
Combined evisceration (yes vs. no)	2.3810	1.0230-5.5034	0.043	0.8485	0.2850-2.5262	0.768
Operation duration, min	1.0033	0.9999-1.0067	0.059	0.9981	0.9932-1.0031	0.464
Estimated blood loss, ml	1.0002	1.0000-1.0004	0.020	1.0000	0.9998-1.0003	0.723
Histology						
DDLS vs. WDLS	6.1106	2.2131-16.8721	<0.001	4.3936	1.3598-14.1964	0.013
Post-operative complications (yes vs. no)	4.2402	1.8759-9.5840	<0.001	2.9746	0.8703-10.1668	0.082
Severe complications (yes vs. no)	2.1589	0.6335-7.3577	0.219	–	–	–
Length of hospital stay, day	1.0269	0.9930-1.0618	0.121	–	–	–
TRL vs. CR	0.4017	0.1714-0.9413	0.036	0.4168	0.1638-1.0609	0.0663

## 4 Discussion

Retroperitoneal liposarcoma (RPLS) is a relatively rare malignant tumor with four distinct histological subtypes. The most common subtypes are well-differentiated liposarcoma and dedifferentiated liposarcoma. Other less common types include myxoid liposarcoma and pleomorphic liposarcoma. Well-differentiated liposarcomas (LPS) are characterized by low-grade malignancy, slow growth, and minimal symptoms. These tumors can reach a substantial size before diagnosis, and achieving R0 resections (complete removal) is often challenging (9). Notably, the rate of local recurrence for retroperitoneal LPS is significantly higher than that for LPS with distant metastasis. In contrast, dedifferentiated LPS can exhibit extreme aggressiveness. Multifocal disease is common in retroperitoneal LPS. At initial presentation, 34% of patients have multifocal disease, and 57% of patients with unifocal disease progress to multifocal disease upon recurrence after chemotherapy or radiation (5, 10, 11). Therefore, when treating patients with retroperitoneal liposarcoma, both the extent of surgical resection and tumor biology must be carefully considered (6).

The current standard of care for treating RPLS involves complete resection (CR). However, CR is associated with a high rate of recurrence, necessitating more extensive resections (12–14). A novel surgical technique, known as total (ipsilateral) retroperitoneal lipectomy (TRL), has emerged. In TRL, the surgeon removes the ipsilateral retroperitoneal adipose tissue en bloc with the tumor, aiming not only for complete resection but also to address multifocal disease while preserving organs rather than performing aggressive resections. Despite being proposed by sarcoma surgeons, clinical studies evaluating the safety and efficacy of TRL remain limited. In our study, patients who underwent TRL surgery showed no significant difference in overall complication rates and rates of severe complications (Grade 3 or higher) compared to patients who underwent traditional CR surgery. These findings demonstrate excellent safety. Furthermore, when compared to other studies, our results also indicate satisfactory safety (15, 16).

In all enrolled patients, TRL significantly improved patients’ recurrence-free survival (RFS) and overall survival (OS). Notably, subgroup analysis revealed that this survival benefit was only present in patients with dedifferentiated liposarcoma (DDLS). This suggests that due to the generally milder and less aggressive nature of WDLS, satisfactory treatment outcomes can be achieved with CR surgery alone. However, it is common for tumors in the same patient to contain both well-differentiated and dedifferentiated components (17). Histologic type has long been considered the most important factor affecting the prognosis of retroperitoneal soft tissue sarcomas, including its impact on overall survival, recurrence-free survival, and distant metastasis (18–20). The impact stems from poorly differentiated sarcomas, known for their high invasiveness and indistinct margins, often infiltrating nearby structures. This can result in microscopic residual tumors at the surgical margin, even when an R0 resection appears successful macroscopically, thereby increasing recurrence risk and reducing disease-free survival. There is currently no established method for effectively assessing pathological margins and the extent of infiltration in retroperitoneal soft tissue sarcomas, either preoperatively or intraoperatively. Surgeons must depend on their experience to determine tumor borders or the depth of invasion into nearby organs, which guides decisions on the extent of resection. The possibility of postoperative complications affects the decision-making process regarding combined organ resection to secure clear margins. For instance, removing organs like the colon, kidney, and psoas generally poses a low risk of severe postoperative complications, whereas resections involving the pancreas, duodenum, or major blood vessels are associated with higher risks of severe complications. Assessing tumor differentiation based solely on imaging and gross examination is unreliable. Therefore, relying solely on imaging to determine the differentiation type and depth of tumor invasion for retroperitoneal liposarcomas (LPS) when deciding between CR or TRL surgery is not feasible (8, 21–23). Studies by Singer et al. indicates that the condition of the surgical margin independently influences the prognosis of RPLS (24–26). Therefore, on the basis of controlling the risk of complications, adopting more aggressive surgical techniques to achieve negative margins becomes essential. Unfortunately, due to the lack of description of margin status in the postoperative pathological results of most patients, we were unable to incorporate and analyze data on margins in this study. Nevertheless, considering the similar safety profiles observed in the study for both surgical approaches, we still recommend TRL surgery for all retroperitoneal LPS cases.

Multifocal disease has profound effects on the oncological outcomes of retroperitoneal liposarcoma (RPLS) patients. In a recent study, 20% of patients presented with multifocal disease, and the 5-year overall survival (OS) rate was significantly lower in the multifocal group than the unifocal group (11). Another study found that 25% of RPLS patients presented with multifocal disease, which was associated with curtailed OS (23). Additionally, a clinical study that included both primary and recurrent RPLS cases showed that the proportion of multifocal disease at initial presentation was 45% (23% for primary cases and 22% for first-recurrent RPLS). Interestingly, the 3-year OS rate after TRL was significantly higher than the 3-year OS rate after CR in patients with multifocal disease (27). Our own research findings indicate that although TRL did not demonstrate improved RFS and OS compared to CR surgery for multifocal liposarcomas among primary RPLS patients, it also did not perform worse than CR. Therefore, our results are consistent with the recommendation of TRL for multifocal RPLS based on the previous study.

Our study had several limitations. First, due to its retrospective nature, our study had inherent biases. Second, the low incidence rate of RPLS results in a scarcity of specialized centers dedicated to diagnosing and treating this disease. Patients are often dispersed across various surgical specialties such as gastrointestinal surgery and urology. Conducting standardized, multicenter clinical studies specifically targeting this condition becomes challenging, which in turn limits the number of patients included in our research. Nevertheless, the critical clinicopathological characteristics were comparable between the two groups. Notably, the proportion of patients receiving adjuvant therapy was relatively low. This allowed us to more accurately compare the effectiveness of the two surgical approaches while minimizing interference from nonsurgical therapies.

## 5 Conclusion

Total (ipsilateral) retroperitoneal lipectomy (TRL) is a relatively safe surgical approach for primary retroperitoneal liposarcoma (RPLS) patients. It has been associated with significantly better recurrence-free survival (RFS) and overall survival (OS) in particular subsets of patients. Further clinical research is needed, particularly in experienced sarcoma centers, to design more standardized and larger-scale studies that can validate the therapeutic efficacy of TRL for RPLS.

## Data Availability

The raw data supporting the conclusions of this article will be made available by the authors, without undue reservation.
